# Meiotic dysfunction accelerates somatic aging in *Caenorhabditis elegans*

**DOI:** 10.1111/acel.13716

**Published:** 2022-09-29

**Authors:** Julia A. Loose, Francis R. G. Amrit, Thayjas Patil, Judith L. Yanowitz, Arjumand Ghazi

**Affiliations:** ^1^ Department of Pediatrics, John G. Rangos Sr. Research Center University of Pittsburgh School of Medicine Pittsburgh Pennsylvania USA; ^2^ Department of Obstetrics, Gynecology, and Reproductive Sciences, Magee‐Womens Research Institute University of Pittsburgh School of Medicine Pittsburgh PA USA; ^3^ Department of Developmental Biology, John G. Rangos Sr. Research Center University of Pittsburgh School of Medicine Pittsburgh Pennsylvania USA; ^4^ Department of Cell Biology & Physiology University of Pittsburgh School of Medicine Pittsburgh Pennsylvania USA

**Keywords:** aging, *C. elegans*, germ cells, germline, healthspan, lifespan, meiosis, proteostasis, reproduction, soma‐germline signaling

## Abstract

An expanding body of evidence, from studies in model organisms to human clinical data, reveals that reproductive health influences organismal aging. However, the impact of germline integrity on somatic aging is poorly understood. Moreover, assessing the causal relationship of such an impact is challenging to address in human and vertebrate models. Here, we demonstrate that disruption of meiosis, a germline restricted process, shortened lifespan, impaired individual aspects of healthspan, and accelerated somatic aging in *Caenorhabditis elegans*. Young meiotic mutants exhibited transcriptional profiles that showed remarkable overlap with the transcriptomes of old worms and shared similarities with transcriptomes of aging human tissues as well. We found that meiosis dysfunction caused increased expression of functionally relevant longevity determinants whose inactivation enhanced the lifespan of normal animals. Further, meiotic mutants manifested destabilized protein homeostasis and enhanced proteasomal activity partially rescued the associated lifespan defects. Our study demonstrates a role for meiotic integrity in controlling somatic aging and reveals proteostasis control as a potential mechanism through which germline status impacts overall organismal health.

## Graphical abstract

How reproductive cells impact health and aging of the whole animal is poorly understood. This study demonstrates that disrupting germline integrity by incapacitating the process of meiosis, which only occurs in germ cells, causes reduction in lifespan and health and induces signs of premature aging in the whole animal.

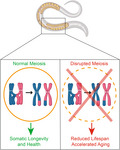

**Abbreviations**
ANM: age‐at‐natural‐menopause
DEG: differentially expressed genes
DSB: double strap break
HR: homologous recombination
POI: premature ovarian insufficiency
WT: wild type

## 1 INTRODUCTION

The impact of increasing maternal age on fertility decline is well documented (Broekmans et al., 2009) but how germline integrity influences organismal aging remains poorly understood. In model organisms, sterility is often associated with increased longevity leading to the dogma of an antagonistic relationship between reproductive‐ and somatic‐health (Flatt, 2011). However, studies in worms, flies, mice and species in the wild have revealed that sterility *per se* does not confer longevity, and reproductive signals also promote lifespan and health (reviewed in Amrit & Ghazi, 2017). Emerging clinical and epidemiological data indicate that reproductive defects augur detrimental long‐term health consequences in both sexes (Cedars et al., 2017). In women, early loss of gonadal function, due to premature ovarian insufficiency (POI) or early age‐at‐natural‐menopause (ANM), is linked to greater susceptibility to cardiovascular disease, diabetes, dementia, osteoporosis and death (Muka et al., 2016; Tsiligiannis et al., 2019). Conversely, women with late ANM exhibit younger “epigenetic aging” profiles, individuals with familial history of longevity show delayed reproductive aging and there are increasing evidences of “rejuvenating” effects of pregnancy (Falick Michaeli et al., 2015; Levine et al., 2016; Perls et al., 1997). Thus, a compelling body of clinical evidence demonstrating that germline status influences organismal senescence is transforming the germline–soma relationship from a domain of evolutionary theory to a topic of significant biomedical relevance. However, correlative human studies neither test causality nor reveal the mechanisms by which the immortal germline may alter the aging of the mortal somatic tissues. Previous studies, including ours, have used the nematode, *Caenorhabditis elegans*, to dissect the impact of germline signals on lifespan, predominantly relying on sterile, long‐lived mutants (Amrit & Ghazi, 2017).

Here, we chose to perturb the function of genes involved in meiosis, a germline‐specific process critical in the sexual life cycle of all eukaryotes for the production of haploid gametes (Hillers et al., 2017), to directly test a causal relationship between germline integrity and somatic aging. Our data show that loss‐of‐function mutations in meiotic genes shortened lifespan, impaired individual aspects of healthspan and proteostasis and induced transcriptional profiles reminiscent of aging worms and human tissues. Enhancing proteostasis partially rescued the lifespan reduction driven by meiosis dysfunction providing mechanistic insights into the influence of meiotic status on somatic health.

## 2 MUTATIONS IN GENES INVOLVED IN ALL ASPECTS OF MEIOSIS SHORTEN LIFESPAN

**FIGURE 1:**
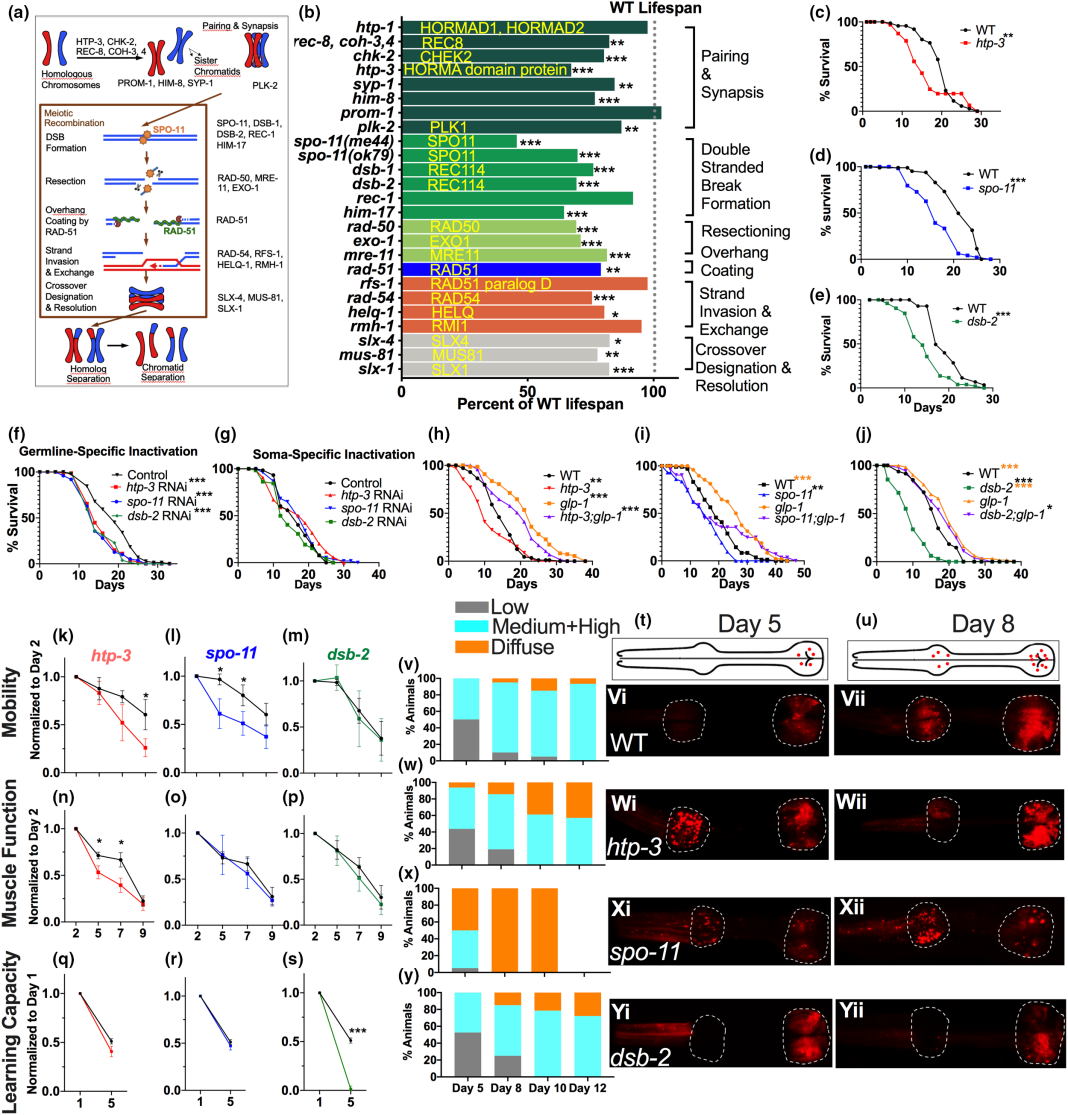
Mutations in meiotic genes shorten lifespan, impair individual aspects of healthspan and disrupt proteostasis. (a) Overview of *C. elegans* meiosis with key genes regulating different steps. (b) Genes with lifespan‐shortening mutations identified here are grouped into color bars based on the step they function in (indicated on the right). Bars indicate percent reduction in lifespan of wild‐type (WT) animals (black hashed line) and associated human homologs where known. (c–e) Lifespans of *htp‐3* (red), *spo‐11* (blue) and *dsb‐2* (green) mutants compared to WT (black). (c) WT (*m* = 19.8 ± 0.5), *htp‐3* (*m* = 16.4 ± 0.8, *p* 0.0014). (d) WT (*m* = 19.4 ± 0.6), *spo‐11* (*m* = 16.3 ± 0.6, *p* 0.0008). (e) WT (*m* = 19.5 ± 0.5), *dsb‐2* (*m* = 14.8 ± 0.5, *p* < 0.001). (f–j) Germline‐specific inactivation of meiosis genes shortens lifespan. (f, g) Germline‐specific RNAi strain DCL569 [*rde‐1; sun‐1p::rde‐1*] (Zou et al., 2019) (f) or soma‐specific RNAi strain NL3511 [*ppw1*(*pk1425*) (Tijsterman et al., 2002) (g) grown from egg stage onward on empty control vector (Control, Ctrl, black) or subjected to RNAi of *htp‐3* (red), *spo‐11* (blue) or *dsb‐2* (green). (f) Ctrl (*m* = 18.9 ± 0.8), *htp‐3* RNAi (*m* = 15.7 ± 0.4, *p* vs. Ctrl <0.0001), *spo‐11* RNAi. (*m* = 15.0 ± 0.4, *p* vs. Ctrl <0.0001), *dsb‐2* (*m* = 15.3 ± 0.4, *p* vs. Ctrl <0.0001). (g) Ctrl (*m* = 16.6 ± 0.6), *htp‐3* RNAi (*m* = 17.2 ± 0.1.0, *p* vs. Ctrl 0.4), *spo‐11* RNAi (*m* = 17.4 ± 0.7, *p* vs. Ctrl = 0.58), *dsb‐2* (*m* = 14.9 ± 0.7, *p* vs. Ctrl 0.2). (h–j) Impact of meiotic mutations on *glp‐1*. (orange) longevity. (h) WT (m = 14.7 ± 0.5), *glp‐1* (m = 22.1 ± 0.6), *htp‐3* (*m* = 11.6 ± 0.6, *p* vs. WT <0.0001), *htp‐3;glp‐1* (*m* = 19.0 ± 0.6, *p* vs. WT <0.0001, *p* vs. *glp‐1* < 0.0009). (i) WT (*m* = 19.9 ± 0.7), *glp‐1* (*m* = 26.5 ± 1.2), *spo‐11* (*m* = 15.0 ± 1.0, *p* vs. WT 0.0005), *spo‐11;glp‐1* (*m* = 20.2 ± 1.9, *p* vs. *glp‐1* 0.09). (j) WT (*m* = 16.5 ± 0.5), *glp‐1* (*m* = 19.8 ± 0.6), *dsb‐2* (*m* = 9.6 ± 0.7, *p* vs. WT <0.0001), *dsb‐2;glp‐1* (*m* = 18.3 ± 0.6, *p* vs. *glp‐1* 0.13). (k–yii) Meiotic mutants show accelerated age‐related decline in mobility (k–m), muscle function (n–p), associative learning capacity (q–s) and proteostasis loss (t–yii). (k–m) Rate of mobility decline measured as the number of thrashes in the liquid between Days 2 to 9 of adulthood. The deficits of *htp‐3* and *spo‐11* mutants are not due to developmental defects as they show normal mobility as pre‐adult L4 larvae (Figure S1E). (n–p) Rate of decline in muscle function measured as the number of pharyngeal pumps between Days 2 to 9 of adulthood. (q–s) Rate of decline in associative learning capacity, measured by testing the ability to associate the odor with food, in adults between Days 1 and 5 of adulthood. (t–yii) Proteostasis phenotypes. (t, u) Cartoon depiction of tagRFP::PAB‐1 aggregates (red dots) in the pharynx of Day 5 (t, few aggregates restricted to posterior bulb) and Day 8 (u, aggregates seen in the anterior bulb and increased in the posterior bulb). Comparison of aggregates in pharyngeal bulbs (outlined in white) of WT (v–vii), *htp‐3* (w–wii), *spo‐11* (x–xii) and *dsb‐2* (y–yii) mutants, with representative images of Day 5 (vi, wi, xi, yi) and Day 8 (vii, wii, xii, yii) animals, and quantifications for Days 5, 8, 10 and 12 in v, w, x and y, respectively. Graphs also indicate a fraction of animals showing diffused and disordered fluorescence (orange) rarely seen before Day 10 in WT but became visible by Day 5 in >50% and ~100% of *htp‐3* and *spo‐11* mutants, respectively. In c–j, data were analyzed using Kaplan Meier statistics and shown as mean lifespan (*m*) ± standard error. Details and data from additional trials are in Tables S2C–E and S3F–J. In k–yii, data are from 3 or 4 trials (≥20 animals/strain/trial). t–yii show data from one of three trials with similar results. Statistical significance was calculated using unpaired two‐tailed (KM) or one‐tailed (n–s) *t*‐tests. Asterisks indicate statistical significance of <0.05 (*), <0.001 (**), <0.0001 (***) and the color of the asterisks indicates the strain/condition being compared to.

We measured the lifespans of 38 strains carrying mutations in genes operating at various stages of meiosis (Hillers et al., 2017). Thirty‐one exhibited a statistically significant lifespan reduction in at least one trial and 13 were short‐lived in at least two trials (Figure 1a,b, Table S1). Since many genes involved in double‐stranded break (DSB) induction and homologous recombination (HR) during meiosis also act in HR and DNA repair in mitotic somatic cells (Marcon & Moens, 2005), *C. elegans* is uniquely suited for addressing their meiotic roles as its adult somatic tissues are post‐mitotic and many, though not all, meiotic genes exhibit germline‐enriched or germline‐specific expression (Han et al., 2019; Hillers et al., 2017). Even so, we asked if this explained the meiotic mutants' short lifespans. But, 9/13 genes had no roles in DNA repair and the others showed disparate effects on DSB induction as measured by DNA localization of the RAD‐51 recombinase (Alpi et al., 2003; Mets & Meyer, 2009); RAD‐51 foci were absent, or normal or displayed altered temporal dynamics in different short‐lived mutants. Similarly, no unifying correlation was seen between germline apoptosis or the extent of fertility defects with lifespan reduction (Table S2).

We chose three genes for detailed investigation based on their meiosis‐specific roles and degree of lifespan effects: *spo‐11* (encodes ortholog of human SPO11 and initiator of meiotic DSBs; Dernburg et al., 1998), *htp‐3* (encodes conserved HORMA‐domain protein that links DSB formation to homolog pairing and synapsis; Goodyer et al., 2008) and *dsb‐2* (encodes protein with structural homology to human REC‐114 that is required for efficient DSB induction; Rosu et al., 2013). Multiple *spo‐11* alleles as well as *htp‐3* and *dsb‐2* mutants exhibited an average lifespan reduction of 25%, 33% and 20%, respectively, over independent trials (Figure 1c–e, Table S1). Germline‐restricted RNAi (Zou et al., 2019) of *dsb‐2*, *htp‐3*, and *spo‐11* throughout life was sufficient to shorten lifespan significantly, whereas, soma‐specific RNAi (Tijsterman et al., 2002) had no effect (Figure 1f, g, Table S3A,B) nor did RNAi during adulthood only (Figure S1A, Table S3C). Using the temperature‐sensitive sterile, *glp‐1*, mutant (Arantes‐Oliveira et al., 2002), we found that *dsb‐2* mutation expectedly did not shorten *glp‐1* lifespan but *htp‐3* mutation induced a small reduction and *glp‐1;spo‐11* showed a biphasic lifespan curve with higher early deaths (Figure 1h–j, Table S3D). *spo‐11*, *dsb‐2* and *htp‐3* have been reported to be germline restricted (Dernburg et al., 1998; Goodyer et al., 2008; Reinke et al., 2004; Rosu et al., 2013) and we did not detect any somatic expression of *spo‐11* either (Figure S1B). Thus, while somatic roles of *spo‐11* and *htp‐3* cannot be overruled, *spo‐11*, *htp‐3* and *dsb‐2* inactivation in the germline was sufficient to shorten lifespan.

## 3 MEIOTIC MUTANTS EXHIBIT A PREMATURE DECLINE IN INDIVIDUAL ASPECTS OF HEALTHSPAN AND PROTEOSTASIS

Young *spo‐11* mutants resembled aging wild‐type (WT) worms in appearance (Garigan et al., 2002; Figure S1C,D) hence we examined the healthspan of meiotic mutants (Keith et al., 2014). Age‐linked mobility loss (measured as the rate of reduction in the animal “thrashing” in liquid) occurred significantly earlier and was more pronounced in *spo‐11* and *htp‐3* mutants compared to WT, but not in *dsb‐2* (Figures 1k–m). Age‐related loss of muscle function (measured as the rate of reduction in pharyngeal‐muscle pumping) was significantly accelerated in *htp‐3* mutants between Days 2 and 5 compared to WT, while *spo‐11* and *dsb‐2* mutants showed similar but smaller effects (Figure 1n–p). In *C. elegans*, age‐related neurological dysfunction is measured by assessing the progressive decline in associative learning capacity with age (Kauffman et al., 2010; Vohra et al., 2018). By Day 5, while ~50% of WT adults retained learning capacity, none of the *dsb‐2* mutants did; *htp‐3* mutants performed a little worse than WT while *spo‐11* behaved normally (Figure 1q–s).

We tested for premature loss of proteostasis, a conserved molecular hallmark of aging (Lopez‐Otin et al., 2013), by examining protein aggregation dynamics in a strain expressing an RFP‐tagged protein, PAB‐1, that shows age‐linked aggregation (Figure 1t–yii; Lechler et al., 2017). *spo‐11* and *htp‐3* mutants manifested significantly higher and earlier protein aggregation while *dsb‐2* had a modest impact (Figure 1w–yii). We detected a small fraction of WT adults wherein fluorescence appeared diffused rather than as distinct punctae. While this was rarely seen before Day 10 in WT, a major fraction of the three meiotic mutants showed it at earlier ages (Figure 1v,w,x,y). Using another proteostasis marker, a temperature‐sensitive *unc‐52* mutant that undergoes whole‐body paralysis at high temperature and is an established reporter of protein folding efficiency (Ben‐Zvi et al., 2009), we found that meiotic genes' inactivation increased paralysis considerably (Figure S1F). Thus, no healthspan feature tested was affected in all three mutants and none showed deficits in every feature tested. Altogether, these results indicated a compromised protein homeostasis environment in meiotic mutants and impairment of individual aspects of healthspan.

### 3.1 Enhancing proteasomal function rescues lifespan defects of *spo‐11* mutants

We compared the transcriptional profiles of Day 1 adult *dsb‐2*, *htp‐3*, and *spo‐11* mutants with age‐matched WT animals. 7654 genes were upregulated (SPO11‐UP) and 3584 downregulated (SPO11‐DOWN) in *spo‐11* mutants. 4730 genes were upregulated (HTP3‐UP) and 1666 downregulated (HTP3‐DOWN) in *htp‐3* mutants. In *dsb‐2* mutants, 106 genes were upregulated (DSB2‐UP) and 91 downregulated (DSB2‐DOWN; Figure S2A–D, Table S4A–F). Notably, *htp‐3* mutants shared 86% of their transcriptome with *spo‐11* mutants (4069/4730 UP, 1430/1666 DOWN; *p* < 0.0001; Figure S2A,B), and despite the small number of differentially expressed genes (DEGs) in *dsb2*, a striking 63 of 106 DSB2‐UP genes (59%, *p* < 0.0001) were also upregulated in *both spo‐11* and *htp‐3* mutants (Figure S2C; Table S4G).

**FIGURE 2:**
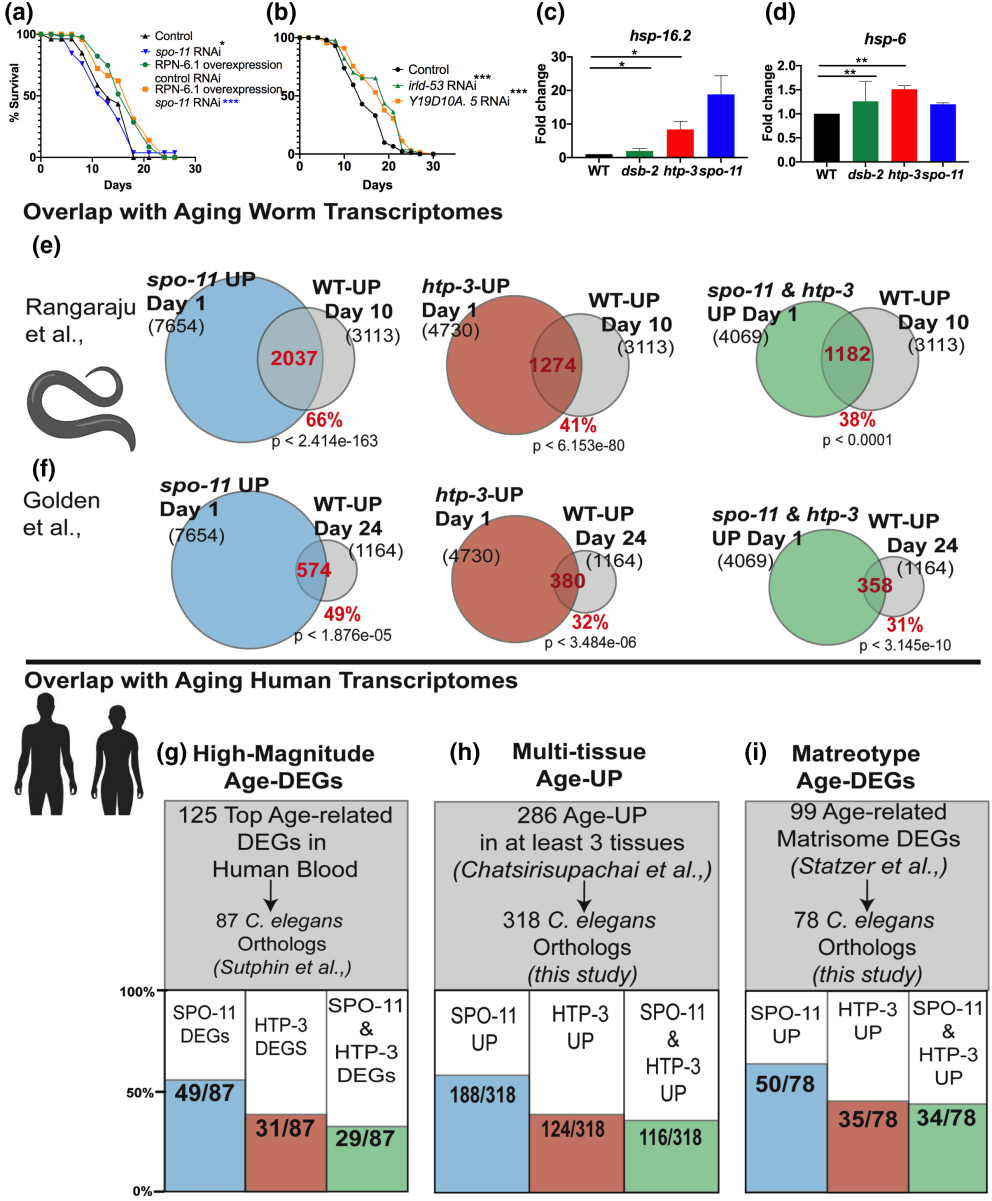
Meiotic mutants show similarities with transcriptomes of aging animals. (a) RPN‐6.1 overexpression rescues *spo‐11* mutants' lifespan. Transgenic control strain on the empty vector (Control, Ctrl, black, *m* = 14.4 ± 0.6) or *spo‐11* RNAi (blue, *m* = 12.7 ± 0.6, *p* 0.0403). RPN‐6.1 overexpressing strain on control vector (green, *m* = 16.6 ± 0.4) or *spo11* RNAi (orange, *m* = 16.8 ± 0.5, *p* vs. control strain on *spo‐11* RNAi <0.0001). (b) RNAi of genes upregulated in meiotic mutants increases wild‐type (WT) lifespan. Control RNAi (Ctrl, black, *m* = 14.8 ± 0.4); *irld‐53* (green, *m* = 17.9 ± 0.6, *p* vs. Ctrl <0.0001); *Y19D10A.5* (orange, *m* = 17.8 ± 0.6, *p* vs. Ctrl 0.0001). (c, d) Expression of chaperone genes in meiotic mutants. Q‐PCR analysis comparing mRNA levels of *hsp‐16.2* (c) and *hsp‐6* (d) in WT vs. mutants. In a and b, survival data analyzed using Kaplan Meier statistics and shown as mean lifespan (m) ± standard error. Details and data from additional trials are in Tables S5A and S6B. In c and d, data were combined from three independent biological replicates, each including three technical replicates. Error bars indicate standard error. (e–i) Meiotic mutants show premature aging transcriptional profiles. Overlap between genes upregulated in young *spo‐11* (blue), *htp‐3* (red) mutants or both (green) with genes upregulated in aged *C. elegans* (gray, e, f) and aging human tissues (g–i). (e, f) Overlap with genes upregulated in Day 10 WT adults in Rangaraju et al. (2015). (e) or Day 24 in Golden et al. (2008) (f). (g–i) Overlap with the *C. elegans* orthologs of genes exhibiting highest magnitude age‐related expression change in human blood (g), upregulated with age in at least three human tissues (h), or the human “matreotype,” the age‐linked expression profile of extracellular matrix or “matrisome” genes (i). Details in Tables S7. *p < 0.05, **p < 0.001, ***p < 0.0001.

In light of their proteostasis phenotypes, it was notable that genes downregulated in the meiotic mutants included proteostasis factors known to promote longevity including (i) *rpn‐6.1*, encoding a 19S proteasome subunit that enhances lifespan in germline‐less animals (Vilchez et al., 2012) and (ii) *cct‐8* and *cct‐2*, chaperone‐encoding genes that modulate somatic and germline proteostasis (Noormohammadi et al., 2016; Samaddar et al., 2021; Vilchez et al., 2012). RPN‐6.1 overexpression partially rescued *spo‐11* mutants' short lifespan but CCT‐2 or CCT‐8 overexpression did not (Figure 2a, Figure S3A, Table S5). Thus, enhancing proteasomal activity mitigated some negative impacts of meiotic dysfunction suggesting that premature loss of somatic proteostasis may partially underlie their longevity deficits.

The upregulated DEGs were enriched for genes encoding transmembrane and cytoskeletal proteins, stress response factors and genes involved in somatic longevity pathways such as insulin/IGF1 (*irld‐35*, and *irld‐53*), TGFβ (*unc‐2*), TOR (*F39C12.1*) and DAF‐12 signaling (*Y19D10A.5*; Templeman & Murphy, 2018; Figure S3B, Table S4G).

We tested if these DEGs were functionally relevant or simply biomarkers of aging. Indeed, whole‐life (but not adult‐only) RNAi of *irld‐53* (encoding a putative insulin binding protein) and *Y19D10A.5* (encoding a putative transmembrane protein) significantly enhanced the lifespan of WT animals (Figure 2b, Table S6), whereas RNAi of *C01B4.7* (encoding a sugar transporter) had significant but inconsistent lifespan enhancements across trials (Table S6). *spo‐11* and *htp‐3* also showed premature upregulation of chaperone genes operating in specific stress‐response pathways (Higuchi‐Sanabria et al., 2018): *hsp‐16.2* and *hsp‐6*, representing the heat‐shock response and mitochondrial unfolded protein response, respectively, were upregulated, but *hsp‐4* representing the endoplasmic reticulum unfolded protein response, was not (Figure 2c,d, Figure S3C).

## 4 *spo‐11* and *htp‐3* mutants show similarities with transcriptomes of aging animals

Based on their aging phenotypes, we hypothesized that the meiotic mutants may exhibit a prematurely aged transcriptional profile, i.e., during young adulthood show high expression of genes normally upregulated in old WT animals. We used *spo‐11* and *htp3* DEGs to test this given their high overlap and the small number of *dsb‐2* DEGs. Comparing the transcriptomes of Day 1 *spo‐11* and *htp‐3* mutants with those of middle‐aged (Day 5) and aging (Day 10) WT animals (Rangaraju et al., 2015) revealed a striking overlap. 66% of genes upregulated on Day 10 in WT were upregulated in Day 1 *spo‐11* mutants (2037/3113, *p* < 2.4e−163), 41% were upregulated in *htp‐3* (1274/3113, *p* < 6.1e−80) and 38% were elevated in both (1182/3113, *p* < 5.0e−96; Figure 2e, Table S7A). The overlap with Day 5 WT transcriptome was also highly significant: 55% (1413/2548, *p* < 5.1e−40) and 42% (1069/2548, *p* < 3.4e−72) for SPO11‐UP and HTP3UP gene lists, respectively (Figure S4A–C, Table S7A). Similarly, genes downregulated in Day 1 *spo‐11* and *htp‐3* mutants strongly overlapped with genes whose expression diminished by Day 5 and Day 10 in WT animals (Figure S4D–I, Table S7A). We obtained similar results upon comparisons with other studies elucidating aging worm transcriptomes. A 50% (574/1164, *p* < 1.8e−05) of genes that Golden et al. found to be upregulated throughout lifespan up to Day 24 were included in the SPO11‐UP class, 33% (380/1164, *p* < 3.4e−06) in the HTP3‐UP class and 31% (358/1164, *p* < 3.145e−10) in both (Figure 2f, Table S7B; Golden et al., 2008). Thus, meiotic mutants' lifespan reduction appeared to be accompanied by signs of a premature somatic aging transcriptional profile.

Recently, Sutphin et al. (2017) screened the Cohorts for Heart and Aging Research in Genomic Epidemiology (CHARGE) consortium's expression datasets to enumerate the top 125 genes with the highest magnitude of age‐related differential expression in blood, and identified 87 *C. elegans* genes orthologous to this group. Remarkably, 51/87 were included in *spo‐11* or *htp‐3* DEGs (Figure 2g, Table S7C). This led us to ask if the meiotic mutants showed similarities with human‐aging transcriptomes from other tissues as well. Previously, Chatsirisupachai et al. examined data from 26 human tissues in the Genotype‐Tissue Expression (GTEx) project to enumerate genes upregulated with age in 10 tissues (Chatsirisupachai et al., 2019). We analyzed these data to look for genes shared between multiple aging tissues and identified 286 genes (“Aging‐DEGs”) upregulated in at least 3 of the 10 tissues (Figure S5, Table S7D). We found 318 *C. elegans* genes orthologous to 135 of the 286 (the larger number of worm genes is due to widespread gene duplications seen in the worm genome; Kim et al., 2018; Woollard, 2005). Of the 318 worm orthologs, 188 were upregulated in *spo‐11* (59%, *p* < 1.0e−08), 124 in *htp‐3* (39%, *p* < 1.3e−06) and 116 in both (36%, *p* < 4.0e−08; Figure 2h, Table S7D). Lastly, we examined the 99 genes comprising the human aging “Matreotype” (age‐linked expression profile of extracellular matrix or “matrisome” genes; Statzer et al., 2021) and found 78 (orthologs of 59 genes) encoded in the worm genome. Of these, 50 (64%, *p* < 1.8e−04), 35 (45%, *p* < 4.5e−04) and 34 (44%, *p* < 4.8e−05) were upregulated in *spo‐11*, *htp‐3* or both mutants, respectively (Figure 2i, Table S7E). Thus, the transcriptional profiles of meiotic mutants showed similarities with multiple gene‐expression profiles associated with human aging.

Altogether, using the unique strengths of *C. elegans*, our study provides direct evidence for the impact of meiosis on the health and longevity of the whole organism. This not only substantiates the close links between reproductive and somatic fitness but also the broader influence of germline integrity on organismal aging. Many of the genes we examined have human homologs with roles in mammalian meiosis (Hillers et al., 2017; Kim et al., 2018) and have been implicated in ANM and/or POI, including 5/13 factors whose mutants showed shortened lifespan (EXO1, HELQ1, CHEK2, RAD‐54, and RAD‐51; Jiao et al., 2018). The transcriptional similarities we identified between meiotic mutants and aging human tissues suggest avenues to unravel potential evolutionarily conserved mechanisms underpinning the meiotic control of health and longevity.

## AUTHOR CONTRIBUTIONS

AG conceived the project. AG, JLY and JAL designed the experiments; JAL, FRGA, TP and AG performed the experiments; AG and JAL wrote the manuscript with input from the other authors.

## CONFLICT OF INTEREST

The authors declare no conflict of interest.

## Supporting information


Figure S1

Figure S2

Figure S3

Figure S4

Figure S5

Appendix S1

Table S1

## Data Availability

The data that supports the findings of this study are available in the Supplementary Material of this article.
