# Supplementary figures and images for: Meiotic dysfunction accelerates somatic aging in Caenorhabditis elegans
Source: Aging Cell. 2022 Sep 29;21(11):e13716. doi: 10.1111/acel.13716 (PMC9649607; doi:10.1111/acel.13716)

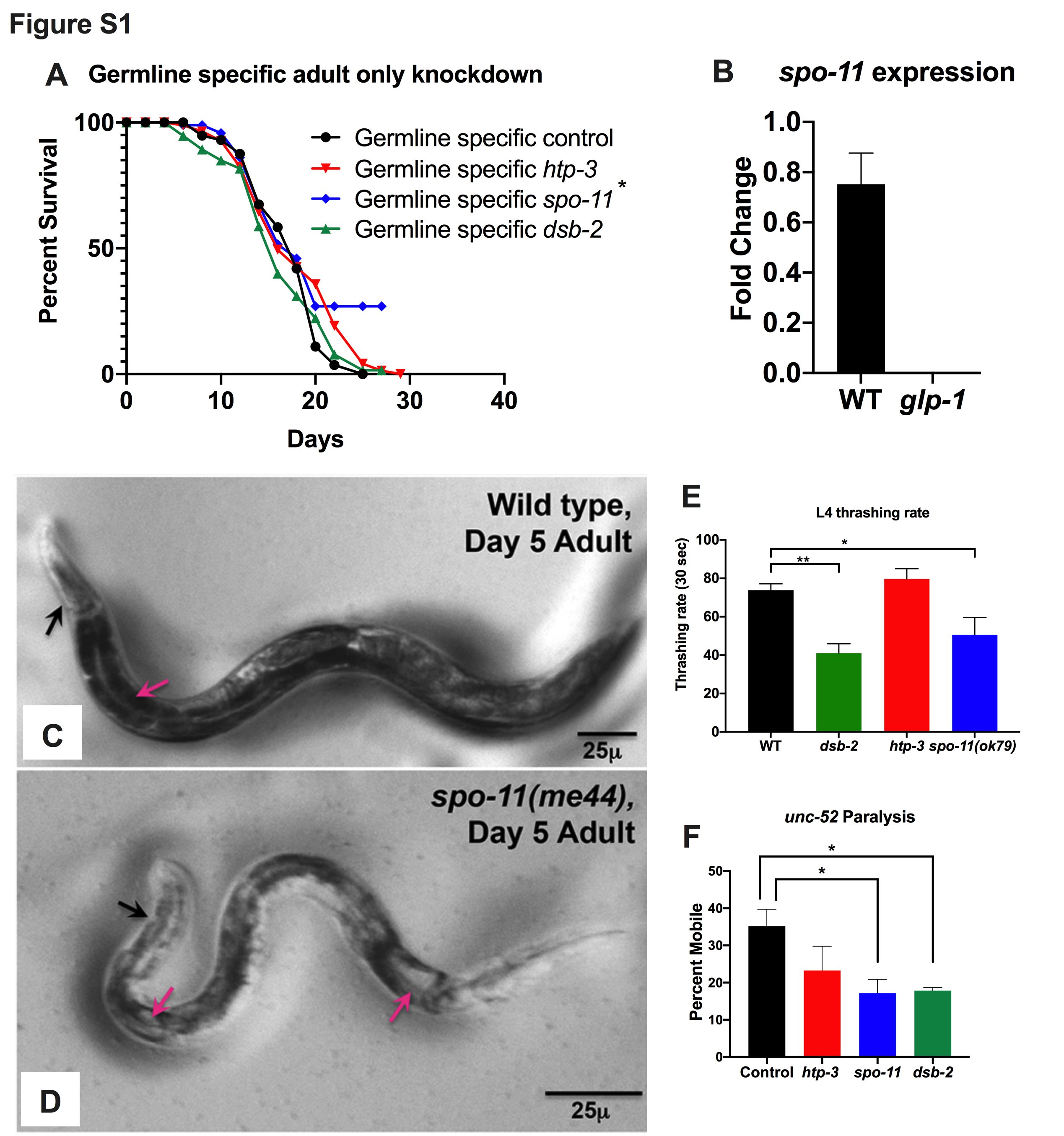

Supplement: Supplementary file 1 — Figure S1 [file ACEL-21-e13716-s004.tiff]

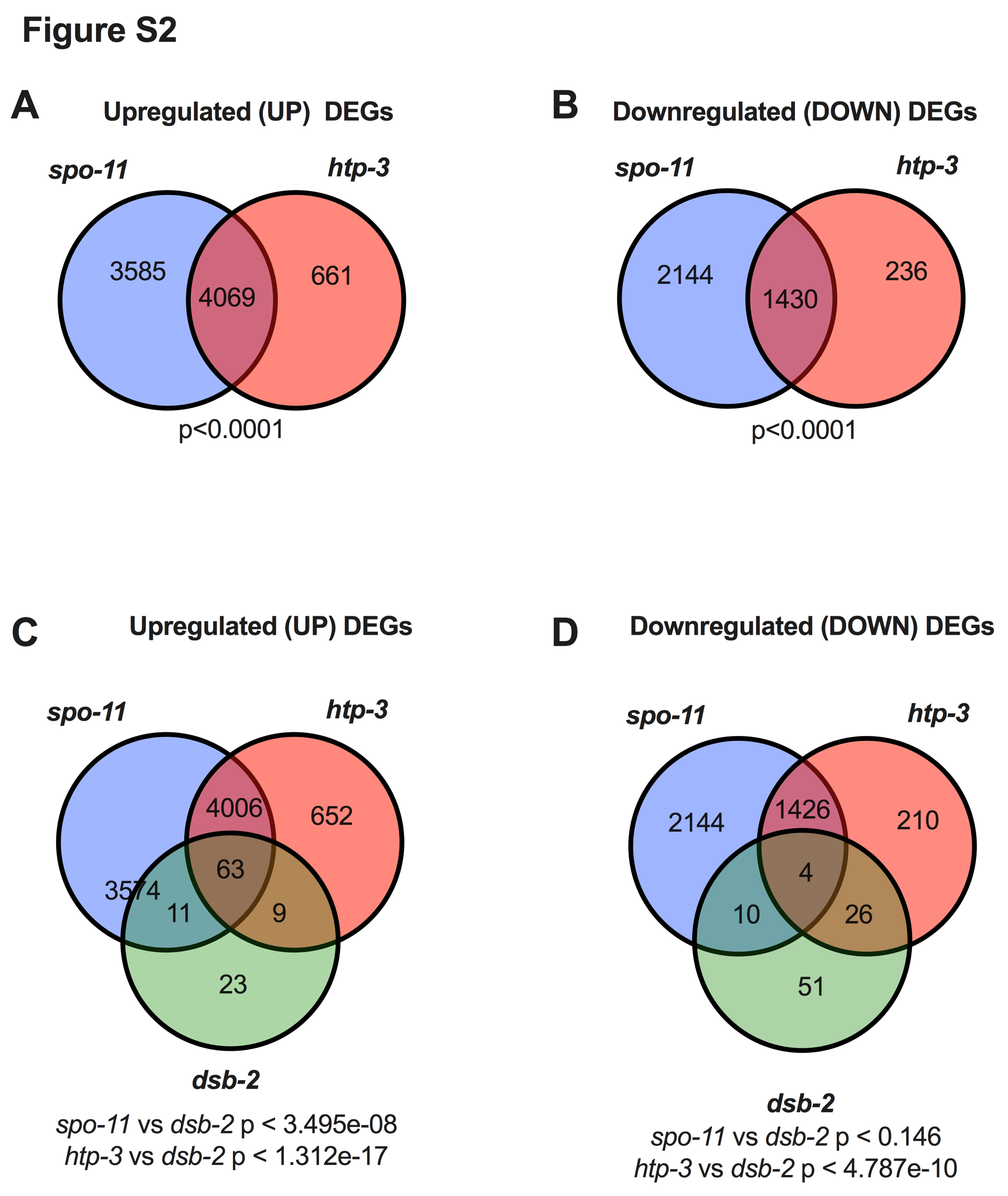

Supplement: Supplementary file 2 — Figure S2 [file ACEL-21-e13716-s003.tiff]

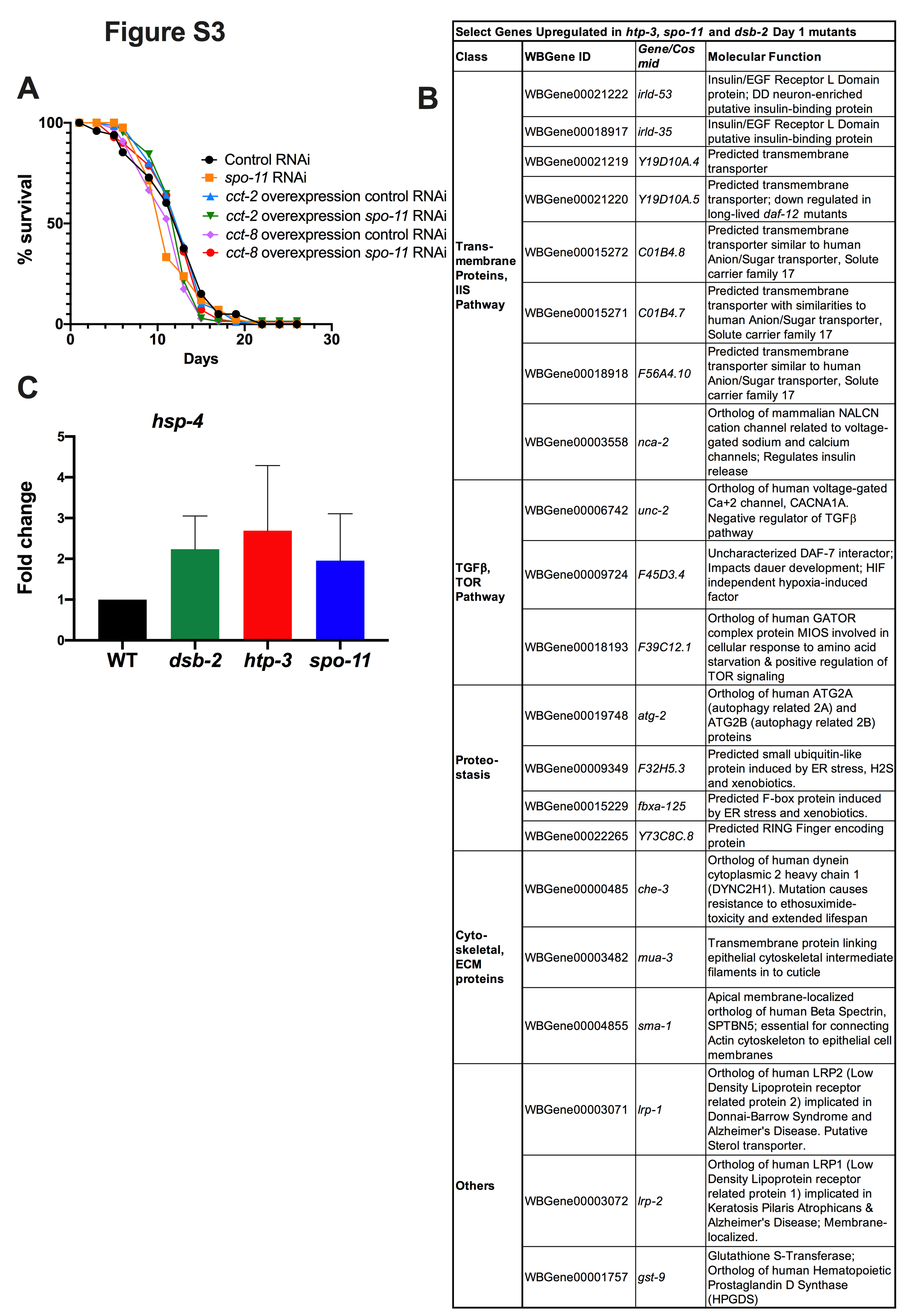

Supplement: Supplementary file 3 — Figure S3 [file ACEL-21-e13716-s001.tiff]

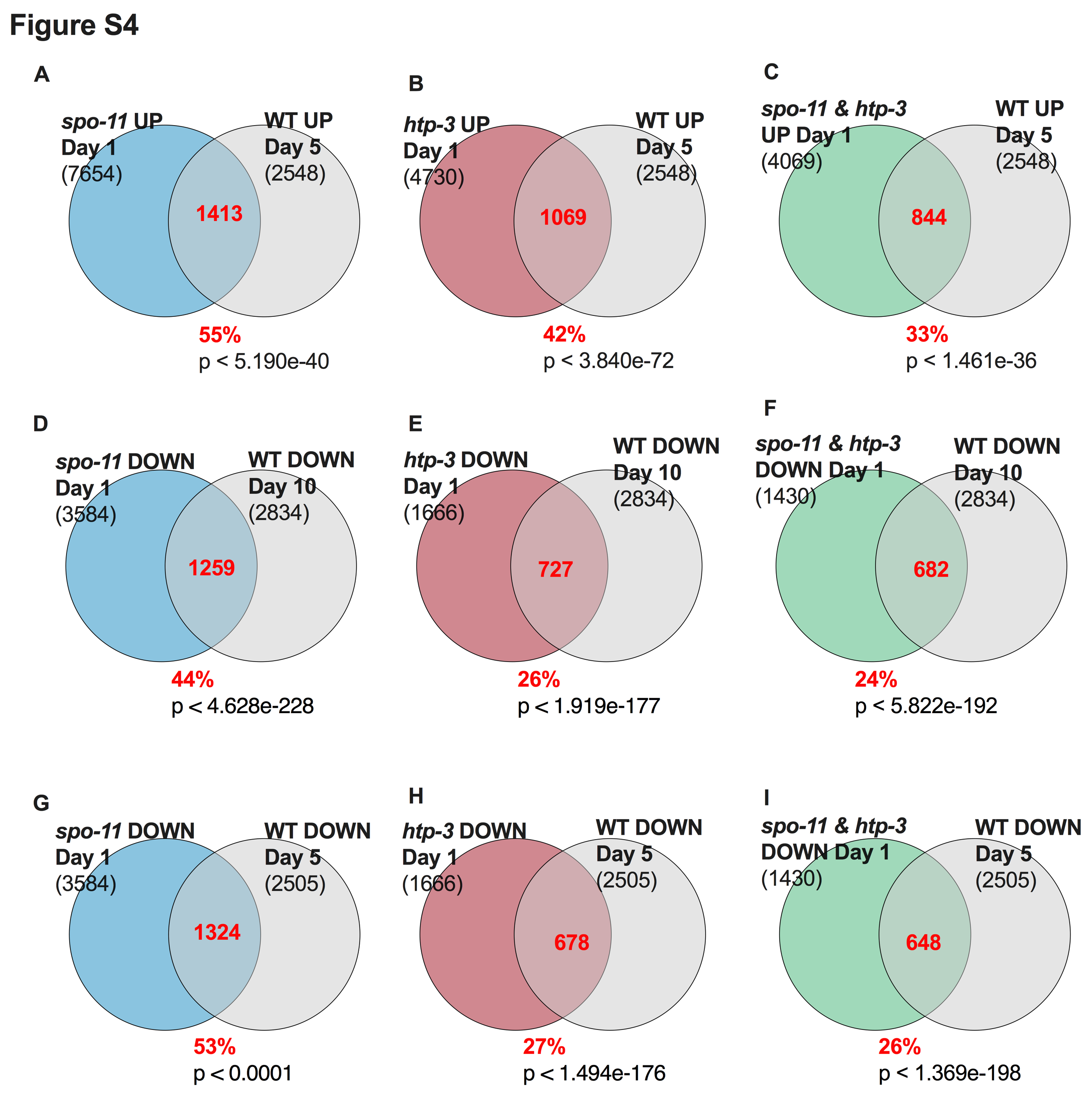

Supplement: Supplementary file 4 — Figure S4 [file ACEL-21-e13716-s002.tiff]
