# Supplementary material for: Meiotic dysfunction accelerates somatic aging in Caenorhabditis elegans
Source: Aging Cell. 2022 Sep 29;21(11):e13716. doi: 10.1111/acel.13716 (PMC9649607; doi:10.1111/acel.13716)
Supplement: Supplementary file 5 — Figure S5 [file ACEL-21-e13716-s007.pdf]

Fig. S5: Overlap of genes differentially expressed in *spo-11* and *htp-3* mutants with worm orthologs of genes identified by Chatsirisupachai et al., 2019 as being differentially expressed with age in different human tissues.

Mutant Strain	↓ Differentiall Expressed Genes in mutants (vs. WT) Category	Number	Adipose (237 UP, 217 DOWN)	Brain (816 UP, 584 DOWN)	Muscle (386 UP, 89 DOWN)	Ovary (343 UP, 422 DOWN)	Uterus (670 UP, 647 DOWN)	Prostate (622 UP, 217 674)
spo-11	**UPregulated**	7654	**136/237 (57%)**	**441/816 (54%)**	**222/386 (58%)**	**178/343 (52%)**	**314/670 (47%)**	**335/622 (54%)**
	**DOWNregulated**	3584	**24/217 (11%)**	**57/584 (10%)**	**18/89 (20%)**	**40/422 (9%)**	**71/647 (11%)**	**80/674 (12%)**
htp-3	**UPregulated**	4730	**101/237 (43%)**	**308/816 (38%)**	**156/386 (40%)**	**119/343 (35%)**	**222/670 (33%)**	**229/622 (37%)**
	**DOWNregulated**	1666	**14/217 (6%)**	**28/584 (5%)**	**7/89 (8%)**	**27/422 (6%)**	**39/647 (6%)**	**41/674 (6%)**
