# Supplementary material for: Meiotic dysfunction accelerates somatic aging in Caenorhabditis elegans
Source: Aging Cell. 2022 Sep 29;21(11):e13716. doi: 10.1111/acel.13716 (PMC9649607; doi:10.1111/acel.13716)
Supplement: Supplementary file 6 — Appendix S1 [file ACEL-21-e13716-s006.pdf]

Meiotic Dysfunction Accelerates Somatic Aging in *C. elegans*

Julia A Loose, Francis RG Amrit, Thayjas Patil, Judith L Yanowitz and Arjumand Ghazi

Supplementary Information

1. **Methods**
2. **Supplementary Discussion**
3. **List of Supplementary Figures**
4. **List of Supplementary Tables**
5. **Supplementary Figure Legends**
6. **Supplementary References**

METHODS

Strains:

Strains were maintained at 20°C on nematode growth media (NGM) plates seeded with *E. coli* strain OP50. Some strains were acquired from the CGC and some generously shared by the Vilchez and Chen labs. These include N2 (WT), AGD614 uthEx633 [*myo-3p::GFP*], AGD598 uthEx557 [*sur-5p::rpn-6* + *myo-3p::GFP*], AGD597 uthEx556 [*sur-5p::rpn-6* + *myo-3p::GFP*], DVG9 *ocbEx9[myo3p::GFP],* DVG47 *ocbEx47[psur5::cct-2, pmyo3::GFP].* DCL569 *mkcSi13 [sun-1p::rde-1::sun-1 3'UTR* + *unc-119(+)] II,* NL355 *ppw-1(pk1425),* CF1903 *glp-1(e2144),* AGP285 *spo-11(ok79)/nT1;glp-1,* AGP304 *dsb-2(me96);glp-1,* AGP322 *htp-3;glp-1,* WS3455 *chk-2(gk212),* QP1298 *rec-8/nT1;coh-3,4,* QP0455 *htp-1(gsk150), htp-1(gk174);htp-2(tm2543), htp-2(t2543),* TY4986 *htp-3(y428) ccIs4251 I/hT2 [bli-4(e937) let-?(q782) qIs48] (I,III),* CA1230 *htp-3(tm3655)/hT2,* AV307 *syp-1(me17)/nT1g,* AC276 *syp-2(ok307)/nT1,* CV2 *syp-3(ok758)/hT2,* CA998 *ieDf2/mLs11,* CA151 *him-8(me4),* RB1183 *prom-1(ok1140),* RB1582 *plk-2(ok1936),* AV157 *spo-11(me44)/nT1,* AV106*, spo-11(ok79)/nT1,* RB1562 *him-5(ok1896),* CA1117 *dsb-1(we11)/nT1g,* QP938 *dsb-2(me96),* KR5301 *rec-1(h2875),* CB5423 *him-17(e2707),* AV473 *rad-50(ok197),* SSM72 *exo-1(tm1842),* QP0900 *mre-11(iow)/nTg,* CA538 *rad-51(lg8701)/nTg, brc-1(tm1842),* RB1279 *rfs-1(ok1372),* VC531 *rad-54(ok615),* TG1792 *helq-1(tm2134),* QP1208 *sws-1(ea12), rmh-1(ad92), him-14(ok230)/mln1,* AV115 *msh-5(me23)/nTiU,* CV98 *him-18(tm2181)III/qCq dpy-19(e1259)glp-1(q339)nls189 III,* TG1760 *mus-81(tm1937),* TG1868 *slx-1(tm2644),* VC193 *him-6(ok412),* CB1487 *xpf-1(e1487).* For strains that are balanced, animals were maintained as heterozygotes and homozygotes were picked for experiments.

Lifespans

All lifespan experiments were conducted at 20°C on *E. coli* OP50 plates, unless otherwise noted. For each strain, between 20 and 30 L4 hermaphrodites were transferred to each of 4-5 plates. For experiments involving RNAi, NGM plates were supplemented with 1mL 100mg/mL Ampicillin and 1mL 1M IPTG (Isopropyl-β-D-1-thiogalactopyranoside) per liter of NGM. Fertile strains were transferred every other day to fresh plates. The plates were observed at 24−48h intervals to record live, dead or censored (animals that were missing or bagged). For lifespan assays that included strains with the temperature sensitive *glp-1* mutant, eggs were picked and transferred to 25°C for 48h. Then the L4 animals were transferred to fresh plates and maintained at 20°C. RNAi strains were acquired from Ahringer or Vidal Library (Kamath et al., 2003; Rual et al., 2004). Kaplan Meier analysis of lifespan data and statistical significance (Mantel Cox) was performed using Online Application of Survival Analysis (OASIS 2 (S. K. Han et al., 2016). GraphPad prism (version 9) was used to graph the results.

Thrashing Assay

To measure thrashing rate with age, L4-stage larvae were picked and maintained at 20°C. On day 2, adults were transferred to an unseeded NGM plate (to remove excess OP50 from the body of the animal), then transferred one at a time into 1ml of M9 media in an unseeded 3cm plate. The animals were allowed to acclimate for 5minutes in the liquid, then the number of body bends counted for a 30 second interval. A body bend constituted the movement of the head and/or tail beyond the midline of the body. Thrashing rate was measured similarly on animals aged to 5, 7, and 9 days of

adulthood at 20°C. Thrashing was quantified for 10-20 animals per timepoint per strain and repeated in 3 independent biological replicates. P values were calculated for each timepoint between the mutant strain and the wild-type strain using a Student's t test.

Pharyngeal Pumping Assay

To measure the decline in pharyngeal pumping rate with age, L4-stage larvae were picked and maintained at 20°C. On day 2, about 20 adults were transferred to a fresh NGM plate seeded with OP50. The animals were allowed to acclimate for about 2-5 minutes. The number of pumps in the terminal bulb was counted for 30 seconds using a counter for every 5 pumps. The count was repeated on the same worm 3 times. Pumping rate was measured similarly on animals aged 5, 7, and 9 days of adulthood at 20°C. Pumping was quantified for 10-20 animals per timepoint per strain and then repeated for a total of 3 biological replicates. Both strains were normalized to the average number of pumps in 30 seconds, for that biological replicate for Day 2. P values were calculated for each time point between the mutant strain and the wild-type strain using Student's t test.

Associative Learning Assay

Chemotaxis response was measured as previously described (Kauffman et al., 2010; Vohra et al., 2018) on a sample size of 20-40 worms per trial and was repeated three times. L4 larvae were picked and maintained at 20°C until D1 of adulthood. The population was then split into the trained and untrained control groups. To perform the chemotaxis assay, 20-40 worms were placed at the center of a 10cm plate, and their

migration towards butanone (1μL 1:10 butanone:ethanol) and ethanol control (1μL ethanol) at opposite ends of the plate was recorded. For the pre-training group, this assay was done immediately, after washing the worms in M9 buffer. For the other group, training consists of a short starvation period, 1hour in M9 buffer, and conditioning 50min with food and butanone (1:10 butanone:ethanol). The worms were then washed and kept on plates with food and no butanone for 30 min, followed by the chemotaxis assay. The same protocol was used on Day 5 adults. The chemotaxis index (CI) was calculated for each group as

$$CI = \frac{(\#\ at\ Butanone - \#\ at\ Control)}{(Total\ \# - \#\ at\ Origin)}$$

CI was then used to calculate the learning index for each strain which was compared between Day 1 and Day 5 adults.

$$Learning\ index = Post\ CI - Pre\ CI$$

Statistical significance was calculated using a Student's t test.

PAB-1 Protein Aggregation

The protocol was previously described by Lechler et al (Lechler et al., 2017). Animals were picked at the L4 stage and maintained on OP50 NGM plates at 20°C. On Day 2 of adulthood, animals were imaged using a Leica M250 FA stereoscope to count the number of aggregates per animal. Each animal was assessed as having low (<10 aggregates), medium (>10 aggregates in posterior bulb), high (>10 aggregates in anterior bulb), or diffuse aggregation (aggregates not countable). At least 20 animals per strain per timepoint were examined for each of three independent biological

replicates. For representative images a Nikon A1r confocal microscope was used to take 0.2μm Z -stacks.

qPCRs

RNA was isolated from three biological replicates of Day 1 adults. These animals were picked as L4's and maintained on *E. coli* OP50 plates at 20°C until collected for RNA. On day 1 of adulthood, the animals were washed with M9 three times. TRIzol was added to the rinsed pellet and stored at -80 °C. Samples were freeze thawed 6 times and RNA isolated using a standard phenol chloroform method with ethanol precipitation and quantified using a Nanodrop (Green & Sambrook, 2020). RNA was treated with DNase 1 (Sigma Aldrich) and cDNA was made using the High Capacity RNA-to-cDNA kit (Applied Biosystems). qPCR reactions were made using PowerUP SYBR Green Master mix (Thermo Fisher), 0.25M primers, and 10ng cDNA and conducted using the CFX Connect Real-Time PCR detection system.

***unc-52* Paralysis Assay**

The assay was adapted from Ben-Zvi et al., (Ben-Zvi et al., 2009). *unc-52(e669su250)* animals were maintained at 15°C till L4 stage then moved to RNAi or control plates at 20 °C to monitor for paralysis. For each experiment, 50-110 animals were used per condition, and the experimenter was blinded to the identity of the RNAi clone. Paralysis was monitored every 4 - 12 hours by prodding the animal with a pick. If the animal did not immediately respond and move across the plate, it was determined to be paralyzed.

GraphPad prism (version 9) was used to graph the results and a student's t-test was used to calculate the statistical significance.

Generation of RNAi Clones

To generate RNAi clones to knockdown *dsb-2* and *irld-53,* PCR was used to amplify a 500bp fragment of each gene. The PCR product was run on a gel, the single band excised and purified using QIAquick gel extraction kit (ID: 28704). The fragment was then ligated into the L4440-TA vector using the NEBNext Quick Ligase Reaction kit (cat # E6056S). After verifying the sequence, the plasmid was transformed into HT115.

RNA Sequencing

Day 1 adults of wild type, *dsb-2(me96), htp-3(y428),* and *spo-11(me44)* mutants (12 samples from 3 independent biological replicates) were harvested in Trizol (Invitrogen) and stored at -80°C. To isolate RNA, the animals were freeze-thawed 6 times, and RNA harvested using the TRIzol method (Green & Sambrook, 2020). RNA quality and concentration were tested using Agilent Tapestation and Qubit Fluorometry. Sequencing libraries were prepared using the NEBNext Poly(A) mRNA Magnetic Isolation module (E7490) and NEBNext Ultra II RNA Library Prep Kit for Illumina (E7775) with NEBNext Multiplex Oligos for Illumina. The libraries were checked for quality and quantified using Tapestation D1000 and Qubit High Sensitivity. Sequencing was performed on Illumina NextSeq 500 with 75 bp single end reads at the Health Sciences Sequencing Core at UPMC Children's Hospital of Pittsburgh.

Transcriptome Analyses

Sequencing data was analyzed using CLC Genomics Workbench (version 21) using the RNA-Seq pipeline. Differentially regulated genes were filtered for significance using a fold change of greater than 2 and a p value <0.05.Enrichment analysis was conducted for each gene group using Wormbase Gene Set Enrichment Analysis tool https://wormbase.org/tools/enrichment/tea/tea.cgi. http://nemates.org/MA/progs/representation.stats.html was utilized to determine the statistical significance of overlap between data sets of differently expressed genes. This program takes into account the size of the gene lists in both sets, the degree of overlap and the size of the *C. elegan*s genome. To identify the *C. elegans* orthologs of human genes, Ortholist (http://ortholist.shaye-lab.org/), (W. Kim et al., 2018) a widely dataset with exhaustive meta analysis of worm-human ortholog conservation (Carlston et al., 2021; Reza et al., 2022; Shemesh et al., 2021) was used.

SUPPLEMENTARY DISCUSSION

The Disposable Soma Theory of Aging posited an antagonistic relationship between the mortal somatic tissues and the immortal germline based on resource trade-off between procreation and organismal maintenance (Kirkwood, 1977). Studies in laboratory animals and in nature have provided support for this premise (Flatt, 2011). But, innumerable exceptions and contrary relationships exist that cannot be explained by a simplistic trade-off (Amrit & Ghazi, 2017; Hansen et al., 2013; Partridge et al., 2005). This dichotomy is epitomized by discoveries in *C. elegans* wherein inducing sterility by removal of the proliferative germline extends lifespan but is reliant entirely upon the presence of intact somatic gonadal tissues and conserved longevity-promoting proteins (Amrit & Ghazi, 2017). In wild species such as eusocial ants, bumblebees and African mole rats, fertile females live longer and healthier lives (Gwynne, 2008; Johns et al., 2018; Negroni et al., 2021; Sahm et al., 2021). Transplantation of ovaries from young mice into older females increases the recipients' lifespan and extends their cardio-protective advantage suggesting beneficial reproductive inputs to the soma (Mason et al., 2009; Mason et al., 2011). Some of the most compelling evidences for the profound impact that the reproductive system has on overall health come from human studies, through clinical and population data linking reproductive aging and gonadal failure with susceptibility to a spectrum of major diseases and death (Jacobsen et al., 2003; Mondul et al., 2005; Ossewaarde et al., 2005; Podfigurna-Stopa et al., 2016; Snowdon et al., 1989; Wu et al., 2014). We attempted to test the impact of germline integrity on somatic fitness directly by focusing on the germline-intrinsic process of meiosis, in a genetically tractable model organism whose unique biology facilitates the investigation of germline

vs. somatic gene functions. Our data suggest that fidelity of meiosis in the germline is crucial for the animal's lifespan and normal rate of aging.

We examined a significant fraction, though not all, of genes involved in worm meiosis (38 strains targeting 35 genes out of 91 reported so far) (Hillers et al., 2017) focusing predominantly on ones with meiosis-specific roles and germline-specific or germline-enriched expression patterns (M. Han et al., 2019; Hillers et al., 2017; Reinke et al., 2004). We chose genes representing the various steps of the process from meiotic entry (*chk-2*), pairing, synapsis and cross over (CO) formation of homologous chromosomes (*plk-2*, *htp-3, syp-3),* induction of double stranded breaks (DSBs) (*spo-11, dsb-2*), meiotic homologous recombination (HR) (*rad-51, helq-1, rad-54)*, CO resolution and chromosome separation (*slx-1*) (Hillers et al., 2017). Mutations in genes acting at every stage we tested showed lifespan reduction and collectively these effects could not be simply explained by other causes such as germline apoptosis (Bohr et al., 2016; Gartner et al., 2000; Stergiou et al., 2007) or DNA damage roles or reproductive output. Many of the short-lived mutants exhibited modest or severe fertility phenotypes, while others had normal or near normal fertility, either because the mutations disrupted a meiotic step that did not impact overall chromosome segregation or due to redundancy or partial loss of function. For instance, mutations in *him-8*, that functions in homolog pairing in the early steps of meiosis, specifically impair X chromosome segregation, and though a large number of mutant embryos are XO males and XXX hermaphrodites, they usually receive correct number of autosomes resulting in high viability, including in *him-8(me4)*, the mutant we tested and that showed ~23% lifespan

reduction (Phillips et al., 2005). Alternatively, the function of the exoribonuclease, EXO-1, in resection of meiotic DSBs is partially dependent on MRE-11, a member of the MRX/N complex (Yin & Smolikove, 2013) and *exo-1* mutants have no significant fertility defects (O'Neil et al., 2013) but showed lifespan reduction. Expectedly though, the larger fraction of mutants had reproductive phenotypes. In fact, given previous observations that sterility and reduced fertility are often associated with increased longevity (Flatt, 2011; Hautekèete et al., 2001; Smith, 1958; Westendorp & Kirkwood, 1998), it was especially conspicuous that 9 of 13 mutants that had short lifespans experienced >80% fertility loss (Table S2). Germline-specific RNAi of meiotic genes reduced lifespan significantly while soma-specific RNAi did not, supporting germline-specific roles of the genes we tested, but, since RNAi induces variable, and often partial reduction of function, this merits caution. Indeed, we noticed smaller lifespan effects upon using RNAi treatments as compared to genetic mutants. To circumvent this, we attempted to utilize the temperature-sensitive sterile, *glp-1* mutant (Amrit & Ghazi, 2017; Arantes-Oliveira et al., 2002) as a complementary approach with equivocal results. *dsb-2* mutation had no impact on *glp-1* longevity as predicted based on germline-specific activity, but *htp-3* and *spo-11* mutations caused some suppression. One confounding aspect of these experiments is the fact that the *glp-1* mutant itself is long lived (Amrit & Ghazi, 2017; Arantes-Oliveira et al., 2002) so, these suppressions may reflect somatic pro-longevity functions of *htp-3* and *spo-11* in this longevity paradigm unrelated to their meiotic roles.

In addition to the diminished lifespans, the three mutants we studied in detail exhibited

(a) premature loss of some aspects of healthspan, and (b) significant overlap with transcriptomes of aging worms and aging human tissues, suggesting that their short lifespans may be a consequence of accelerated somatic aging. Interestingly, no mutation impacted all three healthspan measures tested and no single aspect of healthspan was altered in all mutants. Since we did not test every fitness metric the possibility that the meiotic mutants share a common healthspan deficit cannot be overruled. These ostensibly disparate healthspan phenotypes, along with the distinct effects of the three genes on longevity in germline-less animals, make it challenging to envisage a unified mechanism by which meiosis impacts lifespan. However, similar patterns have been observed in other longevity-regulatory paradigms where some aspects of healthspan correlate with lifespan and others do not in mutants/genes in the same longevity paradigm (Zhou et al., 2011) or that a single factor can promote longevity vs. innate immunity by relatively distinct pathways (Naim et al., 2021). It is also in accordance with recent studies, including ours, that have revealed that lifespan and individual aspects of healthspan can be genetically uncoupled (Amrit et al., 2019; Kew et al., 2020; Zhou et al., 2011),

The high overlap that *spo-11* and *htp-3* transcriptomes showed with each other and with the aging *C. elegans* transcriptomes reported by two independent studies (Golden et al., 2008; Rangaraju et al., 2015) provide support for meiotic mutants undergoing accelerated aging and through shared mechanisms. The small number of *dsb-2* DEGs despite their significant lifespan and healthspan phenotypes was surprising. Pertinently though, the *dsb-2* mutation has been reported to exhibit age-related meiosis defects

(Rosu et al., 2013). Since we sequenced Day 1 animals, transcriptional consequences of more pronounced late-life meiotic defects may have been obscured in our study. It was also notable though that despite their small number, over 50% *dsb-2* DEGs overlapped significantly with the other two mutants' transcriptomes. Further, the transcriptomes of young meiotic mutants not only showed strong resemblance to transcriptomes of old worms, but also shared similarities with human aging DEGs from three independent datasets of genes that exhibit (a) highest magnitude upregulation in one tissue, blood, (b) shared upregulation between multiple tissues, and (c) an age-related 'matreotype'. It is noteworthy that previously the correlation between ANM and epigenetic age was observed in blood alone (Levine et al., 2016), whereas, the meiotic mutants also showed overlap with Age-DEGs shared between at least three tissues or more. Recent whole-genome sequencing endeavors have identified a host of genes implicated in ANM and POI with expectedly a significant overlap between the two (Day et al., 2015; Ruth et al., 2021). Genes involved in meiosis and DNA damage repair (DDR) constitute a large fraction of these loci. This includes many we tested, including five of thirteen genes whose mutants shortened lifespan (EXO1, HELQ1, CHEK2, RAD-54, RAD-51) underscoring the relevance of this work to human aging.

LIST OF SUPPLEMENTARY FIGURES

Fig. S1: Additional lifespan and healthspan phenotypes of meiotic mutants.

Figure S2: Overlap of genes differentially regulated in *spo-11, dsb-2,* and *htp-3* mutants

Figure S3: Molecular features and functional relevance of *spo-11* and *htp-3* DEGs

Fig. S4: Overlap of *spo-11* and *htp-3* mutants' DEGs with transcriptional profiles of aging worms.

Fig. S5: Overlap of genes differentially expressed in *spo-11* and *htp-3* mutants with worm orthologs of human aging genes.

LIST OF SUPPLEMENTARY TABLES

Table S1: Impact of meiotic-gene mutations on wild-type lifespan

Table S2: Fertility and related phenotypes exhibited by short-lived meiotic mutants

Table S3A-D: Impact of germline- and soma-specific inactivation on meiotic genes on lifespan

Table S4A-S4G: Lists of differentially expressed genes in meiotic mutants with GO Term analyses, tissue enrichment analyses and identified human orthologs

Table S5: RPN-6.1 overexpression partially rescues the short lifespan of *spo-11* mutants

Table S6: Impact of inactivation of genes upregulated in meiotic mutants on lifespan of wild-type animals

Table S7A-F: Overlap of genes upregulated in young meiotic mutants with genes upregulated in normal aging worms and aging human tissues

SUPPLEMENTARY FIGURE LEGENDS

Figure S1: Additional lifespan and healthspan phenotypes of meiotic mutants.

A: Germline specific adult only knockdown of meiotic genes does not impact lifespan. Control strain *sun-1p::rde-1* on L4440 (Control empty vector) (m=17.19 ± 0.53, n = 55/62), *htp-3* RNAi (m= 17.94 ± .61, n = 74/106 P vs *sun-1p::rde-1* on L4440 0.0732), *spo-11* RNAi (m=17.43 ± .41, n = 66/107 P vs *sun-1p::rde-1* on L4440 0.0485), or dsb-2 RNAi (m=16.29 ± .52, n = 88/97 P vs *sun-1p::rde-1* on L4440=0.8605). Survival data analyzed using Kaplan Meier curve shown as mean lifespan (m) ± standard error of the mean n=observed/total * $p < 0.05$, ** $p < 0.001$, *** $p < 0.0001$ **B:** Q-PCR analysis detects no *spo-11* mRNA in Day 1 adults of *glp-1* mutants whereas WT, fertile animals show robust expression. Statistical significances calculated using a one-tailed t test. Data combined from 3 independent biological replicates. **C-D:** Brightfield microscopy images of Day 5 wild type animals (C) and Day 5 *spo-11(me44)* mutants (D). Arrows indicate large vacuolar structures seen in mutants' intestine (pink) and head region (black) but not in WT. **E**. Rate of mobility as measured by thrashing rate at L4 larval stage in wild type (WT, black), *dsb-2* (green), *htp-3* (red), *spo-11(ok79)* (blue). *htp-3* and *spo-11* mutants that exhibited greater/rapid decline as adults do not show any defects as pre-adult animals, whereas, *dsb-2* mutants whose thrashing decline was similar to WT show a reduced thrashing rate as L4s. Data is represented as an average from 3 biological replicates, with at least 20 animals/strain/replicate. Error bars indicate the standard error of the mean. P value calculated by a One-Way ANOVA**. F**: Protein-folding efficiency assessed as percentage of temperature-sensitive *unc-52* mutants evading paralysis and retaining mobility upon 48hr exposure to 20°C. Animals subjected to RNAi inactivation

of *htp-3* (red), *spo-11* (blue) or *dsb-2* (green) compared to animals grown on empty control vector (black). Data shown is combined from two biological replicates with 82-99 animals tested per strain per biological replicate. Statistical significance was calculated using one tailed (pumping, memory) or unpaired two-tailed (thrashing) t tests. Asterisks indicate statistical significance <0.05 (*), <0.001 (**), <0.0001 (***). Details of healthspan assays are in Methods section.

Figure S2: Overlap of genes differentially regulated in *spo-11, dsb-2* and *htp-3* mutants.

A, B: Overlap of genes upregulated (A) and downregulated (B) in *spo-11* (blue) and *htp-3* (red) mutants. **C, D:** Overlap between genes upregulated (C) or downregulated (D) in *spo-11*, *dsb-2* (green) and *htp-3* mutants. 59% genes upregulated in *dsb-2* mutants were also upregulated in *spo-11* and *htp-3* mutants. http://nemates.org/MA/progs/representation.stats.html was utilized to determine the statistical significance of overlap (shown below each comparison) between data sets of differently expressed genes. Gene lists in Table S4.

Figure S3: Molecular features and functional relevance of *spo-11* and *htp-3* DEGs. A: Overexpression of CCT-2 and CCT-8 does not rescue the lifespan phenotype of *spo-11* mutants. Control strain on grown on bacteria expressing the RNAi control empty vector (black, m=12.39 ± 0.65 n = 40/50) or *spo-11* RNAi (orange, m=11.95 ± 0.5, n = 42/43, P vs Control strain on control RNAi = 0.3896). Overexpression of *cct-2* on RNAi control empty vector (blue, m=12.96 ± 0.29, n = 104/140, P vs control strain on control RNAi = 0.6817) or on *spo-11* RNAi (green, m=12.35 ± 0.26, n= 77/110, P vs control strain on control RNAi =3688). Overexpression of *cct-2* on empty RNAi control vector (purple, m=11.55 ± 0.32, n= 89/109 P vs control strain on control RNAi = 3772) or on *spo-11* RNAi (red, m=12.4 ± 0.32, n= 95/143 P vs control strain on control RNAi = 0.7183). Survival data analyzed using Kaplan Meier statistics and shown as mean lifespan (m) ± standard error of the mean. n = observed/total worms. **B: Select genes upregulated in Day1 *htp-3, spo-11* and *dsb-2* mutants.** Genes involved in known longevity pathways, proteostasis and cytoskeletal or membrane localization upregulated included in the group. Complete gene list in Table S4G. **C: Expression of stress response gene, *hsp-4*, in *dsb-2, spo-11,* or *htp-3* mutants compared to wild type (WT).** Q-PCR analysis of mRNA levels measured in age-matched Day 2 adults. Data combined from three independent biological replicates, each including three technical replicates. Error bars indicate standard error of the mean.

Fig. S4: Overlap of *spo-11* and *htp-3* mutants' DEGs with transcriptional profiles of aging worms.

A-C: Overlap between genes upregulated (UP) in *spo-11* (blue), *htp-3* (red) or both (green) mutants with genes UP in Day 5 wild type (WT) adults in Rangaraju et al. (gray) (Rangaraju et al., 2015). **D-I:** Overlap between genes downregulated (DOWN) in *spo-11* (blue), *htp-3* (red) or both (green) mutants with genes DOWN in Day 10 (D-F) or Day 5 (G-I) WT adults in Rangaraju et al. (gray) (Rangaraju et al., 2015). See Tables S7 for details.

Fig. S5: Transcriptomes of meiotic mutants show overlap with genes upregulated in multiple human aging tissues. Genes identified as being upregulated with age (Age-DEGs) by Chatsirisupachai et al (Chatsirisupachai et al., 2019) in 10 human tissues were analyzed and 286 were found to be shared between 3 tissues or more. DEGs from six tissues shown here (blue boxes) were selected (based on healthspan phenotypes of meiotic mutants, their reproduction relevance and magnitude of differential expression) and their *C. elegans* orthologs identified. These gene lists were compared to the DEGs of *spo-11* and *htp-3* mutants (gray boxes). Percent overlap indicated in each case. Genes upregulated with age showed a high percentage overlap (red) as compared to downregulated ones.

SUPPLEMENTARY REFERENCE

Amrit, F. R. G., & Ghazi, A. (2017). Influences of Germline Cells on Organismal Lifespan and Healthspan. In *Ageing: Lessons from C. elegans* (pp. 109-135).

Amrit, F. R. G., Naim, N., Ratnappan, R., Loose, J., Mason, C., Steenberge, L., . . . Ghazi, A. (2019). The longevity-promoting factor, TCER-1, widely represses stress resistance and innate immunity. *Nat Commun, 10*(1), 3042. doi:10.1038/s41467-019-10759-z

Arantes-Oliveira, N., Apfeld, J., Dillin, A., & Kenyon, C. (2002). Regulation of life-span by germ-line stem cells in Caenorhabditis elegans. *Science, 295*(5554), 502-505. doi:10.1126/science.1065768

Ben-Zvi, A., Miller, E. A., & Morimoto, R. I. (2009). Collapse of proteostasis represents an early molecular event in Caenorhabditis elegans aging. *Proc Natl Acad Sci U S A, 106*(35), 14914-14919. doi:10.1073/pnas.0902882106

Bhalla, N., & Dernburg, A. F. (2005). A conserved checkpoint monitors meiotic chromosome synapsis in Caenorhabditis elegans. *Science, 310*(5754), 1683-1686. doi:10.1126/science.1117468

Bohr, T., Ashley, G., Eggleston, E., Firestone, K., & Bhalla, N. (2016). Synaptonemal Complex Components Are Required for Meiotic Checkpoint Function in Caenorhabditis elegans. *Genetics, 204*(3), 987-997. doi:10.1534/genetics.116.191494

Carlston, C., Weinmann, R., Stec, N., Abbatemarco, S., Schwager, F., Wang, J., . . . Hammell, C. M. (2021). PQN-59 antagonizes microRNA-mediated repression during post-embryonic temporal patterning and modulates translation and stress granule formation in C. elegans. *PLoS Genet, 17*(11), e1009599. doi:10.1371/journal.pgen.1009599

Chatsirisupachai, K., Palmer, D., Ferreira, S., & de Magalhaes, J. P. (2019). A human tissue-specific transcriptomic analysis reveals a complex relationship between aging, cancer, and cellular senescence. *Aging Cell, 18*(6), e13041. doi:10.1111/acel.13041

Chin, G. M., & Villeneuve, A. M. (2001). C. elegans mre-11 is required for meiotic recombination and DNA repair but is dispensable for the meiotic G(2) DNA damage checkpoint. *Genes Dev, 15*(5), 522-534. doi:10.1101/gad.864101

Day, F. R., Ruth, K. S., Thompson, D. J., Lunetta, K. L., Pervjakova, N., Chasman, D. I., . . . Murray, A. (2015). Large-scale genomic analyses link reproductive aging to hypothalamic signaling, breast cancer susceptibility and BRCA1-mediated DNA repair. *Nat Genet, 47*(11), 1294-1303. doi:10.1038/ng.3412 http://www.nature.com/ng/journal/v47/n11/abs/ng.3412.html#supplementary-information

Dernburg, A. F., McDonald, K., Moulder, G., Barstead, R., Dresser, M., & Villeneuve, A. M. (1998). Meiotic recombination in C. elegans initiates by a conserved mechanism and is dispensable for homologous chromosome synapsis. *Cell, 94*(3), 387-398. Retrieved from https://www.ncbi.nlm.nih.gov/pubmed/9708740

Flatt, T. (2011). Survival costs of reproduction in Drosophila. *Exp Gerontol, 46*(5), 369-375. doi:10.1016/j.exger.2010.10.008

Gartner, A., Milstein, S., Ahmed, S., Hodgkin, J., & Hengartner, M. O. (2000). A conserved checkpoint pathway mediates DNA damage--induced apoptosis and cell cycle arrest in C. elegans. *Mol Cell, 5*(3), 435-443. Retrieved from https://www.ncbi.nlm.nih.gov/pubmed/10882129

Golden, T. R., Hubbard, A., Dando, C., Herren, M. A., & Melov, S. (2008). Age-related behaviors have distinct transcriptional profiles in Caenorhabditis elegans. *Aging Cell, 7*(6), 850-865. doi:10.1111/j.1474-9726.2008.00433.x

Goodyer, W., Kaitna, S., Couteau, F., Ward, J. D., Boulton, S. J., & Zetka, M. (2008). HTP-3 links DSB formation with homolog pairing and crossing over during C. elegans meiosis. *Dev Cell, 14*(2), 263-274. doi:10.1016/j.devcel.2007.11.016

Green, M. R., & Sambrook, J. (2020). Total RNA Extraction from Caenorhabditis elegans. *Cold Spring Harb Protoc, 2020*(9), 101683. doi:10.1101/pdb.prot101683

Gwynne, D. T. (2008). Sexual conflict over nuptial gifts in insects. *Annu Rev Entomol, 53*, 83-101. doi:10.1146/annurev.ento.53.103106.093423

Han, M., Wei, G., McManus, C. E., Hillier, L. W., & Reinke, V. (2019). Isolated C. elegans germ nuclei exhibit distinct genomic profiles of histone modification and gene expression. *BMC Genomics, 20*(1), 500. doi:10.1186/s12864-019-5893-9

Han, S. K., Lee, D., Lee, H., Kim, D., Son, H. G., Yang, J. S., . . . Kim, S. (2016). OASIS 2: online application for survival analysis 2 with features for the analysis of maximal lifespan and healthspan in aging research. *Oncotarget, 7*(35), 56147-56152. doi:10.18632/oncotarget.11269

Hansen, M., Flatt, T., & Aguilaniu, H. (2013). Reproduction, fat metabolism, and life span: what is the connection? *Cell Metab, 17*(1), 10-19. doi:10.1016/j.cmet.2012.12.003

Hautekèete, N.-C., Piquot, Y., & Van Dijk, H. (2001). Investment in survival and reproduction along a semelparity–iteroparity gradient in the Beta species complex. *Journal of Evolutionary Biology, 14*(5), 795-804. doi:https://doi.org/10.1046/j.1420-9101.2001.00322.x

Hayashi, M., Chin, G. M., & Villeneuve, A. M. (2007). C. elegans germ cells switch between distinct modes of double-strand break repair during meiotic prophase progression. *PLoS Genet, 3*(11), e191. doi:10.1371/journal.pgen.0030191

Higashitani, A., Aoki, H., Mori, A., Sasagawa, Y., Takanami, T., & Takahashi, H. (2000). Caenorhabditis elegans Chk2-like gene is essential for meiosis but dispensable for DNA repair. *FEBS Lett, 485*(1), 35-39. doi:10.1016/s0014-5793(00)02178-5

Hillers, K. J., Jantsch, V., Martinez-Perez, E., & Yanowitz, J. L. (2017). Meiosis. *WormBook*, 1-43. doi:10.1895/wormbook.1.178.1

Jacobsen, B. K., Heuch, I., & Kvåle, G. (2003). Age at natural menopause and all-cause mortality: a 37-year follow-up of 19,731 Norwegian women. *Am J Epidemiol, 157*(10), 923-929. doi:10.1093/aje/kwg066

Johns, M. E., Warzybok, P., Bradley, R. W., Jahncke, J., Lindberg, M., & Breed, G. A. (2018). Increased reproductive investment associated with greater survival and longevity in Cassin's auklets. *Proc Biol Sci, 285*(1885). doi:10.1098/rspb.2018.1464

Kamath, R. S., Fraser, A. G., Dong, Y., Poulin, G., Durbin, R., Gotta, M., . . . Ahringer, J. (2003). Systematic functional analysis of the Caenorhabditis elegans genome using RNAi. *Nature, 421*(6920), 231-237. doi:10.1038/nature01278

Kauffman, A. L., Ashraf, J. M., Corces-Zimmerman, M. R., Landis, J. N., & Murphy, C. T. (2010). Insulin signaling and dietary restriction differentially influence the decline of learning and memory with age. *PLoS Biol, 8*(5), e1000372. doi:10.1371/journal.pbio.1000372

Kew, C., Huang, W., Fischer, J., Ganesan, R., Robinson, N., & Antebi, A. (2020). Evolutionarily conserved regulation of immunity by the splicing factor RNP-6/PUF60. *Elife, 9*. doi:10.7554/eLife.57591

Kim, W., Underwood, R. S., Greenwald, I., & Shaye, D. D. (2018). OrthoList 2: A New Comparative Genomic Analysis of Human and Caenorhabditis elegans Genes. *Genetics, 210*(2), 445-461. doi:10.1534/genetics.118.301307

Kim, Y., Kostow, N., & Dernburg, A. F. (2015). The Chromosome Axis Mediates Feedback Control of CHK-2 to Ensure Crossover Formation in C. elegans. *Dev Cell, 35*(2), 247-261. doi:10.1016/j.devcel.2015.09.021

Kirkwood, T. B. (1977). Evolution of ageing. *Nature, 270*(5635), 301-304. Retrieved from https://www.ncbi.nlm.nih.gov/pubmed/593350

Lechler, M. C., Crawford, E. D., Groh, N., Widmaier, K., Jung, R., Kirstein, J., . . . David, D. C. (2017). Reduced Insulin/IGF-1 Signaling Restores the Dynamic Properties of Key Stress Granule Proteins during Aging. *Cell Rep, 18*(2), 454-467. doi:10.1016/j.celrep.2016.12.033

Levine, M. E., Lu, A. T., Chen, B. H., Hernandez, D. G., Singleton, A. B., Ferrucci, L., . . . Horvath, S. (2016). Menopause accelerates biological aging. *Proc Natl Acad Sci U S A, 113*(33), 9327-9332. doi:10.1073/pnas.1604558113

Martin, J. S., Winkelmann, N., Petalcorin, M. I., McIlwraith, M. J., & Boulton, S. J. (2005). RAD-51-dependent and -independent roles of a Caenorhabditis elegans BRCA2-related protein during DNA double-strand break repair. *Mol Cell Biol, 25*(8), 3127-3139. doi:10.1128/MCB.25.8.3127-3139.2005

Mason, J. B., Cargill, S. L., Anderson, G. B., & Carey, J. R. (2009). Transplantation of young ovaries to old mice increased life span in transplant recipients. *J Gerontol A Biol Sci Med Sci, 64*(12), 1207-1211. doi:10.1093/gerona/glp134

Mason, J. B., Cargill, S. L., Griffey, S. M., Reader, J. R., Anderson, G. B., & Carey, J. R. (2011). Transplantation of young ovaries restored cardioprotective influence in postreproductive-aged mice. *Aging Cell, 10*(3), 448-456. doi:10.1111/j.1474-9726.2011.00691.x

Mondul, A. M., Rodriguez, C., Jacobs, E. J., & Calle, E. E. (2005). Age at natural menopause and cause-specific mortality. *Am J Epidemiol, 162*(11), 1089-1097. doi:10.1093/aje/kwi324

Naim, N., Amrit, F. R. G., Ratnappan, R., DelBuono, N., Loose, J. A., & Ghazi, A. (2021). Cell nonautonomous roles of NHR-49 in promoting longevity and innate immunity. *Aging Cell, 20*(7), e13413. doi:10.1111/acel.13413

Negroni, M. A., Macit, M. N., Stoldt, M., Feldmeyer, B., & Foitzik, S. (2021). Molecular regulation of lifespan extension in fertile ant workers. *Philos Trans R Soc Lond B Biol Sci, 376*(1823), 20190736. doi:10.1098/rstb.2019.0736

O'Neil, N. J., Martin, J. S., Youds, J. L., Ward, J. D., Petalcorin, M. I., Rose, A. M., & Boulton, S. J. (2013). Joint molecule resolution requires the redundant activities of MUS-81 and XPF-1 during Caenorhabditis elegans meiosis. *PLoS Genet, 9*(7), e1003582. doi:10.1371/journal.pgen.1003582

Ossewaarde, M. E., Bots, M. L., Verbeek, A. L., Peeters, P. H., van der Graaf, Y., Grobbee, D. E., & van der Schouw, Y. T. (2005). Age at menopause, cause-specific mortality and total life expectancy. *Epidemiology, 16*(4), 556-562. doi:10.1097/01.ede.0000165392.35273.d4

Partridge, L., Gems, D., & Withers, D. J. (2005). Sex and death: what is the connection? *Cell, 120*(4), 461-472. doi:10.1016/j.cell.2005.01.026

Phillips, C. M., Wong, C., Bhalla, N., Carlton, P. M., Weiser, P., Meneely, P. M., & Dernburg, A. F. (2005). HIM-8 binds to the X chromosome pairing center and mediates chromosome-specific meiotic synapsis. *Cell, 123*(6), 1051-1063. doi:10.1016/j.cell.2005.09.035

Podfigurna-Stopa, A., Czyzyk, A., Grymowicz, M., Smolarczyk, R., Katulski, K., Czajkowski, K., & Meczekalski, B. (2016). Premature ovarian insufficiency: the context of long-term effects. *J Endocrinol Invest, 39*(9), 983-990. doi:10.1007/s40618-016-0467-z

Rangaraju, S., Solis, G. M., Thompson, R. C., Gomez-Amaro, R. L., Kurian, L., Encalada, S. E., . . . Petrascheck, M. (2015). Suppression of transcriptional drift extends C. elegans lifespan by postponing the onset of mortality. *Elife, 4*, e08833. doi:10.7554/eLife.08833

Reddy, K. C., & Villeneuve, A. M. (2004). C. elegans HIM-17 links chromatin modification and competence for initiation of meiotic recombination. *Cell, 118*(4), 439-452. doi:10.1016/j.cell.2004.07.026

Reinke, V., Gil, I. S., Ward, S., & Kazmer, K. (2004). Genome-wide germline-enriched and sex-biased expression profiles in Caenorhabditis elegans. *Development, 131*(2), 311-323. doi:10.1242/dev.00914

Reza, R. N., Serra, N. D., Detwiler, A. C., Hanna-Rose, W., & Crook, M. (2022). Non-canonical necrosis in two different cell types in a C. elegans NAD+ salvage pathway mutant. *G3 (Bethesda)*. doi:10.1093/g3journal/jkac033

Rinaldo, C., Bazzicalupo, P., Ederle, S., Hilliard, M., & La Volpe, A. (2002). Roles for Caenorhabditis elegans rad-51 in meiosis and in resistance to ionizing radiation during development. *Genetics, 160*(2), 471-479. Retrieved from https://www.ncbi.nlm.nih.gov/pubmed/11861554

Rosu, S., Zawadzki, K. A., Stamper, E. L., Libuda, D. E., Reese, A. L., Dernburg, A. F., & Villeneuve, A. M. (2013). The C. elegans DSB-2 protein reveals a regulatory network that controls competence for meiotic DSB formation and promotes crossover assurance. *PLoS Genet, 9*(8), e1003674. doi:10.1371/journal.pgen.1003674

Rual, J. F., Ceron, J., Koreth, J., Hao, T., Nicot, A. S., Hirozane-Kishikawa, T., . . . Vidal, M. (2004). Toward improving Caenorhabditis elegans phenome mapping with an ORFeome-based RNAi library. *Genome Res, 14*(10B), 2162-2168. doi:10.1101/gr.2505604

Ruth, K. S., Day, F. R., Hussain, J., Martinez-Marchal, A., Aiken, C. E., Azad, A., . . . Perry, J. R. B. (2021). Genetic insights into biological mechanisms governing human ovarian ageing. *Nature, 596*(7872), 393-397. doi:10.1038/s41586-021-03779-7

Sahm, A., Platzer, M., Koch, P., Henning, Y., Bens, M., Groth, M., . . . Dammann, P. (2021). Increased longevity due to sexual activity in mole-rats is associated with transcriptional changes in the HPA stress axis. *Elife, 10*. doi:10.7554/eLife.57843

Saito, T. T., Mohideen, F., Meyer, K., Harper, J. W., & Colaiacovo, M. P. (2012). SLX-1 is required for maintaining genomic integrity and promoting meiotic noncrossovers in the Caenorhabditis elegans germline. *PLoS Genet, 8*(8), e1002888. doi:10.1371/journal.pgen.1002888

Severson, A. F., Ling, L., van Zuylen, V., & Meyer, B. J. (2009). The axial element protein HTP-3 promotes cohesin loading and meiotic axis assembly in C. elegans to implement the meiotic program of chromosome segregation. *Genes Dev, 23*(15), 1763-1778. doi:10.1101/gad.1808809

Shemesh, N., Jubran, J., Dror, S., Simonovsky, E., Basha, O., Argov, C., . . . Yeger-Lotem, E. (2021). The landscape of molecular chaperones across human tissues reveals a layered architecture of core and variable chaperones. *Nat Commun, 12*(1), 2180. doi:10.1038/s41467-021-22369-9

SMITH, J. M. (1958). The Effects of Temperature and of Egg-Laying on the Longevity of Drosophila Subobscura. *Journal of Experimental Biology, 35*(4), 832-842. doi:10.1242/jeb.35.4.832

Snowdon, D. A., Kane, R. L., Beeson, W. L., Burke, G. L., Sprafka, J. M., Potter, J., . . . Phillips, R. L. (1989). Is early natural menopause a biologic marker of health and aging? *Am J Public Health, 79*(6), 709-714. doi:10.2105/ajph.79.6.709

Stamper, E. L., Rodenbusch, S. E., Rosu, S., Ahringer, J., Villeneuve, A. M., & Dernburg, A. F. (2013). Identification of DSB-1, a protein required for initiation of meiotic recombination in Caenorhabditis elegans, illuminates a crossover assurance checkpoint. *PLoS Genet, 9*(8), e1003679. doi:10.1371/journal.pgen.1003679

Stergiou, L., Doukoumetzidis, K., Sendoel, A., & Hengartner, M. O. (2007). The nucleotide excision repair pathway is required for UV-C-induced apoptosis in Caenorhabditis elegans. *Cell Death Differ, 14*(6), 1129-1138. doi:10.1038/sj.cdd.4402115

Vohra, M., Lemieux, G. A., Lin, L., & Ashrafi, K. (2018). Kynurenic acid accumulation underlies learning and memory impairment associated with aging. *Genes Dev, 32*(1), 14-19. doi:10.1101/gad.307918.117

Westendorp, R. G., & Kirkwood, T. B. (1998). Human longevity at the cost of reproductive success. *Nature, 396*(6713), 743-746. doi:10.1038/25519

Wu, X., Cai, H., Kallianpur, A., Gao, Y. T., Yang, G., Chow, W. H., . . . Shu, X. O. (2014). Age at menarche and natural menopause and number of reproductive years in association with mortality: results from a median follow-up of 11.2 years among 31,955 naturally menopausal Chinese women. *PLoS One, 9*(8), e103673. doi:10.1371/journal.pone.0103673

Yin, Y., & Smolikove, S. (2013). Impaired resection of meiotic double-strand breaks channels repair to nonhomologous end joining in Caenorhabditis elegans. *Mol Cell Biol, 33*(14), 2732-2747. doi:10.1128/MCB.00055-13

Zhou, K. I., Pincus, Z., & Slack, F. J. (2011). Longevity and stress in Caenorhabditis elegans. *Aging (Albany NY), 3*(8), 733-753. doi:10.18632/aging.100367
